# Role of exogenous-applied salicylic acid, zinc and glycine betaine to improve drought-tolerance in wheat during reproductive growth stages

**DOI:** 10.1186/s12870-021-03367-x

**Published:** 2021-12-06

**Authors:** Ramadan Shemi, Rui Wang, El-Sayed M. S. Gheith, Hafiz Athar Hussain, Linna Cholidah, Kangping Zhang, Sai Zhang, Longchang Wang

**Affiliations:** 1 College of Agronomy and Biotechnology, Southwest University, Chongqing, 400715 China; 2 Department of Agronomy, Faculty of Agriculture, Cairo University, Giza, 12613 Egypt; 3 Institute of Environment and Sustainable Development in Agriculture, Chinese Academy of Agricultural Sciences, Beijing, 100081 China

**Keywords:** Antioxidant defense mechanisms, Drought-tolerance, Foliar applications, Oxidative stress, Wheat (*Triticum aestivum* L.)

## Abstract

**Background:**

Drought has become a dangerous threat to reduce crop productivity throughout the world. Exogenous applications of regulators, micronutrients, and/or osmoprotectants for inducing drought-tolerance in field crops have been effectively adopted. A controlled pot study was performed to investigate the relative efficacy of salicylic acid (SA), zinc (Zn), and glycine betaine (GB) as foliar applications on the growth, tissues pigments content, relative water content (RWC), leaf gas-exchange, antioxidant enzymes activity, reactive oxygen species (ROS) accumulation, osmolytes contents, and the yield parameters of wheat plants subjected to two soil water conditions (85% field capacity: well-watered, 50% field capacity: water-deficient) during reproductive growth stages.

**Results:**

Water deficient conditions significantly decreased the growth, yield parameters, RWC, photosynthesis pigment, and gas-exchange attributes except for intercellular CO_2_ concentration. However, foliar applications remarkably improved the growth and yield parameters under water deficit conditions. Under drought condition, exogenous applications of SA, Zn, and GB increased the grain yield pot^− 1^ by 27.99, 15.23 and 37.36%, respectively, as compared to the control treatment. Drought stress statistically increased the contents of hydrogen peroxide (H_2_O_2_), superoxide anion radical (O_2_^•−^), and malonaldehyde (MDA), and elevated the harmful oxidation to cell lipids in plants, however, they were considerably reduced by foliar applications. Foliar applications of SA, Zn, and GB decreased MDA content by 29.09, 16.64 and 26.51% under drought stress, respectively, as compared to the control treatment. Activities of all antioxidant enzymes, proline content, and soluble sugar were increased in response to foliar applications under water deficit conditions.

**Conclusions:**

Overall, foliar application of GB, SA, and Zn compounds improved the drought-tolerance in wheat by decreasing the ROS accumulation, promoting enzymatic antioxidants, and increasing osmolytes accumulation. Finally, GB treatment was most effective in thoroughly assessed parameters of wheat followed by SA and Zn applications to alleviate the adverse effects of drought stress.

## Background

Wheat (*Triticum aestivum* L.) is one of the most important cereal crops which is highly susceptible to drought stress [1]. Universally, the wheat crop is the primary vegetable protein source in the diet of humans, containing a higher protein content than other main grain crops such as maize and rice. The increasing population and food demands further necessitate the increase in wheat production to ensure social stability and future food security [2, 3]. In China, wheat is the second-most important food crop after rice and the third-most important crop in total production overall, after maize and rice [4]. The cultivated area of wheat in China was estimated to be 23.73 million ha and it yielded about 133.60 million metric tonnes year^− 1^ [4]. Wheat is sensitive cereal crop to drought stress after maize, especially during the critical growth periods (e.g., flowering stage). However, drought led to the yield reduction among different species, in which maize had higher yield reduction (39.3%) compared to wheat (20.6%) by 40% water deficiency [5]. Drought is the most important one of abiotic stress factors that negatively influence the growth and production of various field crops [6–10]. Drought causes various changes in crop plants through different morphological, physiological, and biochemical responses [11–13]. Many studies have reported that the defense system of antioxidant was increased through the synthesis of antioxidants such as ascorbate peroxidase (APX) and glutathione reductase (GR), and enzymatic antioxidants such as peroxidase (POD), catalase (CAT), and superoxide dismutase (SOD), which were stimulated to quench the ROS production in plants during drought stress [3, 6, 14–16]. Moreover, Datir et al. [17] have reported that the antioxidant enzymes, free proline contents, GB accumulation, MDA, and H_2_O_2_ contents were increased under drought induced by polyethylene glycol (PEG) in wheat cultivars. In addition, Chen et al. [18] remarked that the drought stress slowed down wheat growth and reduced grain yield by impacting the anthesis and grain-filling process, and decreased leaf water potential, stomatal conductance, and the photosynthesis.

Foliar applications of various growth regulators, micronutrients, and osmoprotectants can play the key role in inducing drought-tolerance in plants at various plant growth stages [10, 19–22]. SA is a multifunctional plant hormone that can effectively ameliorate the adverse effects caused by biotic and abiotic stresses [23–26] by modulating different growth responses, and physiological and biochemical characteristics in plants [10, 27–30]. A useful role of the salicylic acid has been noticed in improving drought stress tolerance in wheat by improving the relative water contents, leaf gas-exchange, soluble carbohydrate, proteins, proline contents, the activity of antioxidant enzymes and yield attributes [21, 31, 32]. Furthermore, Zinc is an essential micronutrient that participates in many physiological functions and structure of the regulatory cofactor of many enzymes, carbohydrate, chlorophyll production, pollen function, fertilization, metabolism of RNA, protein synthesis, and the DNA functions [33, 34]. Water deficit condition at grain filling phase statistically reduced the plant height, yield parameters, RWC, chlorophyll contents, and decreased activity of SOD, POD, and CAT, while exogenous applications of zinc and salicylic acid had positive impact on all these parameters and mitigated the harmful effects of water deficit on wheat plants [20, 31, 35]. Rahmani et al. [36] reported that supplemental Zn improved the drought-tolerance in safflower by increasing proline, relative water content, chlorophyll contents, and the yield and its components. Nevertheless, GB has an important role as an osmoprotectants, which help the plants to resist drought through improved leaf gas-exchange and chlorophyll content in maize plants [37]. Wheat genotypes prevent the damage of PEG induced osmotic stress via various mechanisms such as osmolytes accumulation [38]. In addition, net photosynthesis, transpiration rate, and yield attributes were decreased under drought stress at the tillering, flowering, and milking stages in wheat plants, while exogenous application of GB enhanced them under drought treatment [19, 39]. Similarly, Hasanuzzaman et al. [40] recorded that GB played an important role in reducing aggregation and detoxification of ROS, hence recovering photosynthesis and decreasing oxidative stress. Our previous study, Shemi et al. [22] have illustrated that exogenous applications of GB, Zn, and SA substantially improved the activity of CAT, SOD, and APX enzymes, and decreased the contents of MDA and H_2_O_2_, and these changes were beneficial to protect maize leaf tissues from oxidative harm in cell membranes.

However, there is no investigation has been carried out to assess the relative effects of SA, Zn, or GB compounds in inducing drought-tolerance of the wheat crop during reproductive growth stages. The current study was aimed to test the hypothesis that the foliar applications of salicylic acid, zinc, and/or glycine betaine can ameliorate the damaging effects of drought stress in wheat during reproductive growth stages by observing the changes in the scavenging ability of antioxidant defense system, which constitutes the executive for the protective response system. The objectives of the study were to (1) Investigate the response of wheat growth, yield, and its attributes to the exogenous applications of SA, Zn, and GB under different soil water conditions during reproductive growth stages; (2) Determine the effect of respective exogenous applications on chlorophyll contents, RWC, leaf gas-exchange, antioxidant enzymes activity, MDA content, and ROS and osmolytes accumulations under different soil water conditions; and (3) Compare the efficacy of concerned exogenous applications to alleviate the harmful effects of drought stress under different soil water conditions.

## Results

### Growth and yield attributes

Water deficient condition statistically (*p* < 0.05) hampered the wheat growth and yield parameters in terms of plant height, fresh weight of plants pot^− 1^, dry weight of plants pot^− 1^, leaf area of plants pot^− 1^, number of tillers pot^− 1^, number of spikes pot^− 1^, number of grains spike^− 1^, grain weight spike^− 1^, 1000-grain weight, biological yield pot^− 1^, grain yield pot^− 1^, and harvest index in comparison to well-watered condition. However, results showed that growth and yield parameters were improved by SA, Zn, and GB spraying treatments in both stressed and non-stressed plants (Tables 1, 2, and 3). As expected, one or more spraying treatments significantly enhanced all parameters in both soil water conditions except for the number of grains spike^− 1^ as compared to the control. The respective spraying treatments increased plant height by 6.94, 6.92 and 8.05%, fresh weight of plants pot^− 1^ by 18.88, 11.30 and 23.16%, dry weight of plants pot^− 1^ by 15.82, 9.34 and 28.30%, leaf area of plants pot^− 1^ by 39.34, 33.22 and 47.63%, number of tillers pot^− 1^ by 13.07, 8.97 and 15.29%, number of spikes pot^− 1^ by 13.78, 9.72 and 16.33%, number of grains spike^− 1^ by 3.47, 1.23 and 4.95%, grain weight spike^− 1^ by 13.15, 2.26 and 18.42%, 1000-grain weight by 9.75, 4.29 and 13.42%, biological yield pot^− 1^ by 17.63, 12.95 and 25.16%, grain yield pot^− 1^ by 27.99, 15.23 and 37.36%, and harvest index by 8.98, 1.96 and 10.00% under water-deficient condition, respectively, as compared to the values of the control treatment. Overall, maximum growth and yield parameters were recorded from the plants treated with GB followed by SA and Zn spraying treatments as compared with control treatment under both soil water conditions.

**Table 1:** Influences of soil water conditions and foliar treatments on wheat growth parameters

Soil water conditions	Foliar treatments	Plant height (cm)	Fresh weight of plants pot^**− 1**^ (g)	Dry weight of plants pot^**− 1**^ (g)	Leaf area of plants pot^**− 1**^ (cm^**2**^)	No. of tillers pot^**− 1**^	No. of spikes pot^**− 1**^
**WW**	**CK**	63.39^bc^ ± 1.67	73.13^bc^ ± 2.92	17.79^d^ ± 0.73	962.74^c^ ± 54.55	18.62^c^ ± 0.56	16.67^b^ ± 0.33
	**SA**	70.58^a^ ± 1.40	81.20^a^ ± 3.31	21.95^b^ ± 0.61	1067.01^b^ ± 61.24	20.59^ab^ ± 0.55	18.34^a^ ± 0.61
	**Zn**	68.67^ab^ ± 2.64	79.48^ab^ ± 3.62	20.73^bc^ ± 0.73	1015.75^bc^ ± 53.79	19.83^b^ ± 0.60	17.83^b^ ± 0.33
	**GB**	71.86^a^ ± 2.71	82.38^a^ ± 4.11	23.57^a^ ± 0.94	1148.27^a^ ± 65.15	21.27^a^ ± 0.79	19.00^a^ ± 0.34
	**Means**	68.62	79.05	21.00	1048.44	20.07	17.96
**WD**	**CK**	57.73^d^ ± 0.70	43.16^f^ ± 2.38	14.66^f^ ± 0.15	428.14^f^ ± 21.73	14.38^f^ ± 0.61	12.55^d^ ± 0.65
	**SA**	61.74^c^ ± 0.59	51.31^d^ ± 2.04	16.98^e^ ± 0.30	596.60^de^ ± 23.37	16.26^d^ ± 0.53	14.28^c^ ± 0.72
	**Zn**	61.73^c^ ± 0.33	48.04^de^ ± 2.57	16.03^e^ ± 0.72	570.37^e^ ± 27.85	15.67^de^ ± 0.41	13.77^c^ ± 0.63
	**GB**	62.38^bc^ ± 1.70	53.16^d^ ± 2.10	18.81^d^ ± 0.35	632.10^d^ ± 16.41	16.58^d^ ± 0.44	14.60^c^ ± 0.47
	**Means**	60.89	48.92	16.61	556.80	15.72	13.79

Values are means (±SE) of three replicates. For L.S.D.’s results, means with various letters indicate the significant differences (*p* < 0.05). *WW* well-watered; *WD* water-deficient; *CK* control (double distilled water); *SA* salicylic acid; *Zn* zinc; *GB* glycine betaine

**Table 2:** Influences of soil water conditions and foliar treatments on wheat yield and its components

Soil water conditions	Foliar treatments	No. of grains spike^**−1**^	Grain weight spike^**−1**^ (g)	1000-grain weight (g)	Biological yield pot^**− 1**^ (g)	Grain yield pot^**− 1**^ (g)	Harvest index (%)
**WW**	**CK**	38.12^a^ ± 0.97	1.48^bc^ ± 0.03	38.97^bc^ ± 0.93	58.93^cd^ ± 0.73	22.77^cd^ ± 0.73	38.58^b^ ± 2.33
	**SA**	41.40^a^ ± 0.99	1.73^ab^ ± 0.07	41.64^ab^ ± 0.76	67.80^ab^ ± 2.67	27.28^ab^ ± 0.83	40.46^ab^ ± 2.82
	**Zn**	39.42^a^ ± 1.41	1.57^b^ ± 0.03	39.89^b^ ± 0.85	66.32^bc^ ± 3.41	25.42^bc^ ± 0.24	38.62^b^ ± 0.80
	**GB**	42.02^a^ ± 1.71	1.78^a^ ± 0.03	42.52^a^ ± 1.65	70.33^a^ ± 2.61	29.24^a^ ± 0.77	41.70^a^ ± 2.01
	**Means**	40.23	1.64	40.75	65.84	26.18	39.83
**WD**	**CK**	35.69^a^ ± 1.32	1.14^e^ ± 0.04	31.87^f^ ± 0.23	43.16^g^ ± 0.88	15.36^f^ ± 0.29	35.60^cd^ ± 0.74
	**SA**	36.93^a^ ± 1.05	1.29^cd^ ± 0.06	34.98^d^ ± 1.08	50.77^e^ ± 1.71	19.66^de^ ± 0.34	38.80^b^ ± 1.20
	**Zn**	36.13^a^ ± 0.54	1.20^de^ ± 0.04	33.24^ef^ ± 1.39	48.75^ef^ ± 1.39	17.70^e^ ± 0.69	36.30^c^ ± 1.07
	**GB**	37.46^a^ ± 1.12	1.35^c^ ± 0.03	36.15^cd^ ± 0.39	54.02^de^ ± 2.22	21.10^d^ ± 0.53	39.16^ab^ ± 1.50
	**Means**	36.55	1.24	34.05	49.17	18.45	37.46

Values are means (±SE) of three replicates. For L.S.D.’s results, means with various letters indicate the significant differences (*p* < 0.05). *WW* well-watered; *WD* water-deficient; *CK* control (double distilled water); *SA* salicylic acid; *Zn* zinc; *GB* glycine betaine

**Table 3:** *p*-values of the two-way factorial analysis of growth, yield, and physiological and biochemical parameters of wheat as affected by various foliar treatments under both soil water conditions

Parameters	Main factors effects		Interaction effects
	S	F	S × F
**Plant height**	< 0.0001	< 0.0065	< 0.0392
**Fresh weight of plants**	< 0.0001	< 0.0249	< 0.0486
**Dry weight of plants**	< 0.0001	< 0.0001	< 0.0372
**Leaf area of plants**	< 0.0001	< 0.0038	< 0.0435
**Number of tillers**	< 0.0001	< 0.0037	< 0.0467
**Number of spikes**	< 0.0001	< 0.0051	< 0.0245
**Number of grains**	< 0.0004	< 0.1066	< 0.4751
**Grain weight**	< 0.0001	< 0.0001	< 0.0371
**1000-grain weight**	< 0.0001	< 0.0061	< 0.0497
**Biological yield**	< 0.0001	< 0.0007	< 0.0378
**Grain yield**	< 0.0001	< 0.0001	< 0.0212
**Harvest index**	< 0.0073	< 0.0054	< 0.0485
**Chlorophyll a content**	< 0.0087	< 0.0019	< 0.0347
**Chlorophyll b content**	< 0.0180	< 0.0097	< 0.0492
**Total chlorophyll content**	< 0.0163	< 0.0010	< 0.0345
**RWC**	< 0.0001	< 0.0273	< 0.0479
**Net photosynthesis rate**	< 0.0001	< 0.0003	< 0.0198
**Transpiration rate**	< 0.0001	< 0.0008	< 0.0245
**Stomatal conductance**	< 0.0001	< 0.0001	< 0.0359
**Intercellular CO**_**2**_ **concentration**	< 0.0100	< 0.0204	< 0.0457
**APX activity**	< 0.0001	< 0.0002	< 0.0021
**GR activity**	< 0.0001	< 0.0002	< 0.0102
**POD activity**	< 0.0001	< 0.0001	< 0.0260
**CAT activity**	< 0.0001	< 0.0001	< 0.0002
**SOD activity**	< 0.0001	< 0.0001	< 0.0302
**MDA content**	< 0.0001	< 0.0001	< 0.0430
**H**_**2**_**O**_**2**_ **content**	< 0.0001	< 0.0001	< 0.0469
**O**_**2**_^**.-**^ **content**	< 0.0001	< 0.0001	< 0.0041
**Free proline content**	< 0.0001	< 0.0001	< 0.0006
**Total soluble sugar**	< 0.0001	< 0.0001	< 0.0019

*p*-values are considered as significant (*p* < 0.05, *n* = 3) and highly significant (*p* < 0.01, *n* = 3). ‘S’: effect of soil water conditions; ‘F’: effect of foliar treatments; ‘S × F’: effect of the interaction between two variables

### Photosynthetic pigments and RWC

**Fig. 1:**
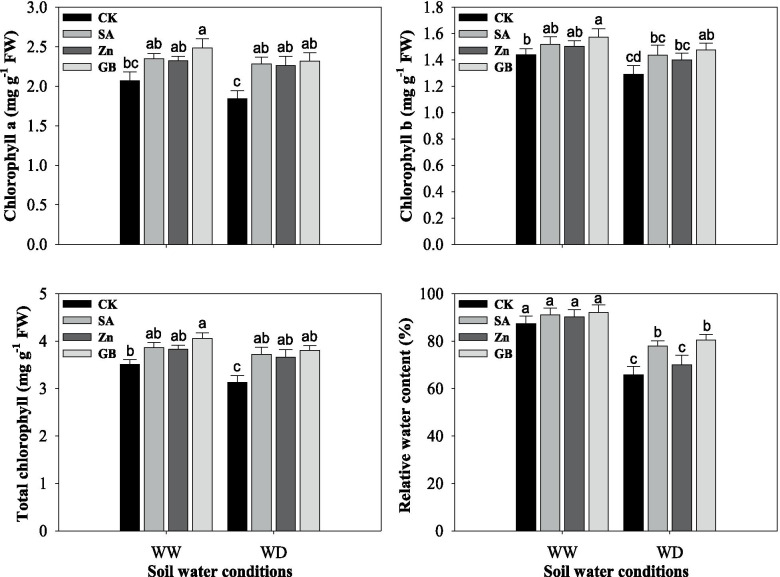
Influences of soil water conditions and foliar treatments on chlorophyll a content, chlorophyll b content, total chlorophyll content and relative water content. Every column in each graph represents the mean (±SE) of three replicates. Various letters above columns indicate the significant differences among means (*p* < 0.05). WW, well-watered; WD, water-deficient; CK, control (double distilled water); SA, salicylic acid; Zn, zinc; GB, glycine betaine

Chlorophyll (*Chl.*) and relative water contents (RWC) of wheat leaves were significantly (*p* < 0.05) decreased by the water-deficient condition. Nevertheless, the *Chl. a*, *Chl. b* and total *Chl.* contents and RWC were enhanced by SA, Zn, and GB spraying treatments in both stressed and non-stressed plants (Fig. 1 and Table 3). The *Chl. a* and total *Chl.* contents were significantly impacted by concerned spraying treatments under water-deficient condition, and GB spraying treatment under well-watered condition, while *Chl. b* content was statistically affected by GB in both soil water conditions and RWC was significantly influenced by SA and GB treatments under water-deficient conditions. Under the water-deficient condition, the respective spraying treatments improved contents of *Chl. a* by 23.91, 22.82 and 26.08%, *Chl. b* by 10.85, 8.52 and 13.95%, total *Chl.* by 18.53, 16.93 and 21.40%, and RWC by 18.55, 6.44 and 22.35%, respectively, as compared to the values of the control treatment. Generally, the highest photosynthetic pigments and RWC were recorded from the plants treated with GB followed by SA and Zn spraying treatments as compared with the control treatment under both soil water conditions.

### Photosynthesis gas-exchange

**Fig. 2:**
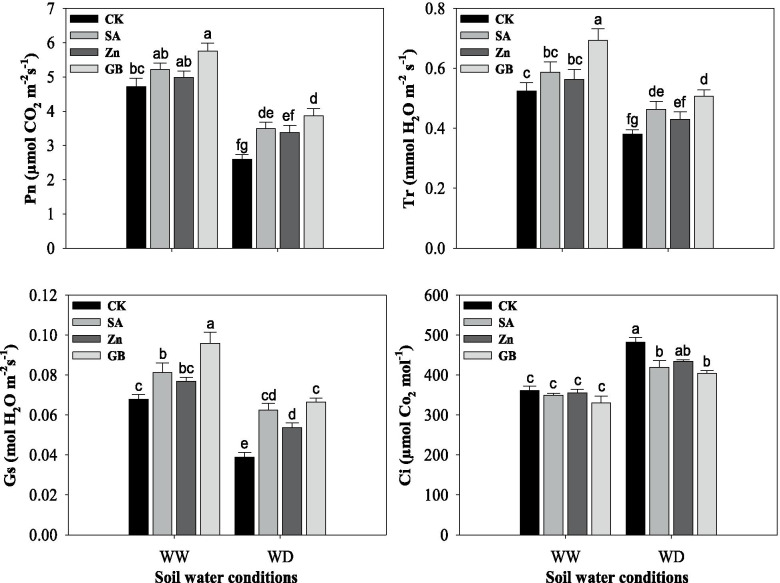
Influences of soil water conditions and foliar treatments on net photosynthesis rate (Pn), transpiration rate (Tr), stomatal conductance (Gs) and intercellular CO_2_ concentration (Ci). Every column in each graph represents the mean (±SE) of three replicates. Various letters above columns indicate the significant differences among means (*p* < 0.05). WW, well-watered; WD, water-deficient; CK, control (double distilled water); SA, salicylic acid; Zn, zinc; GB, glycine betaine

Net photosynthesis rate (Pn), transpiration rate (Tr), stomatal conductance (Gs), and intercellular CO_2_ concentration (Ci) were statistically (*p* < 0.05) influenced by soil water conditions. However, results noticed that photosynthesis gas-exchange parameters were increased by SA, Zn, and GB spraying treatments except for Ci (Fig. 2 and Table 3). Pn and Tr were significantly affected by SA and GB treatments under water-deficient conditions and GB under well-watered conditions, while Gs was considerably impacted by concerned spraying treatments under both soil water conditions except for Zn under the well-watered condition, and Ci was substantially reduced by SA and GB treatments under water-deficient condition. Under the water-deficient condition, the respective spraying treatments promoted the Pn by 34.74, 30.11 and 49.03%, Tr by 21.84, 13.15 and 33.42%, and Gs by 58.97, 35.89 and 69.23%, while they decreased the Ci by 13.01, 9.94 and 16.22%, respectively, as compared to the values of the control treatment. In general, the highest Pn, Tr, and Gs, and the lowest Ci were registered from the plants treated with GB followed by SA and Zn spraying treatments as compared with control treatment under both soil water conditions.

### Enzymatic antioxidants and MDA content

**Fig. 3:**
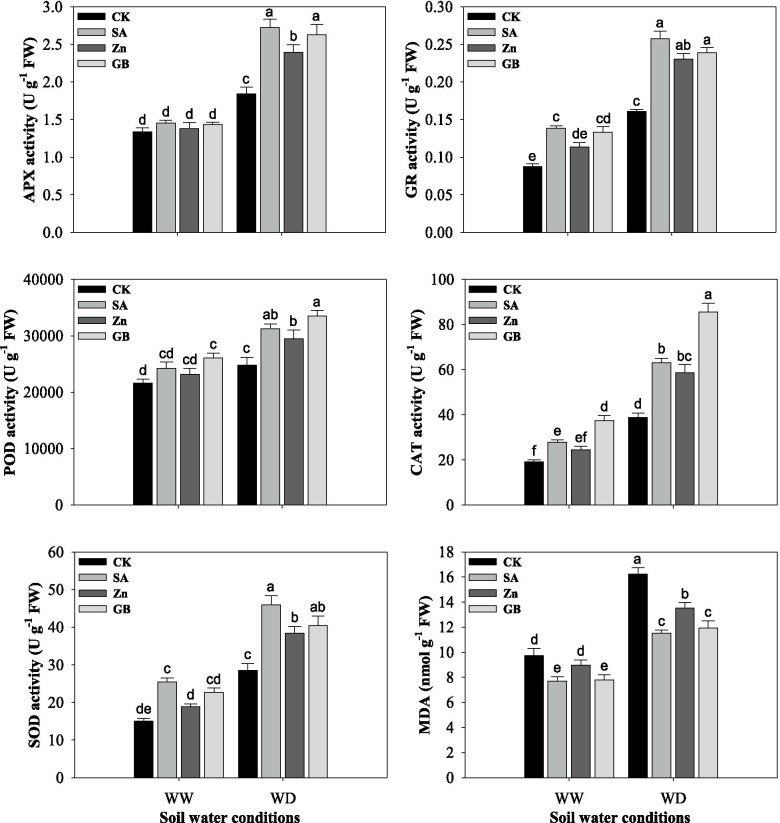
Influences of soil water conditions and foliar treatments on the activities of ascorbate peroxidase (APX), glutathione reductase (GR), peroxidase (POD), catalase (CAT), superoxide dismutase (SOD) and malonaldehyde (MDA) content. Every column in each graph represents the mean (±SE) of three replicates. Various letters above columns indicate the significant differences among means (*p* < 0.05). WW, well-watered; WD, water-deficient; CK, control (double distilled water); SA, salicylic acid; Zn, zinc; GB, glycine betaine

APX, GR, POD, CAT, and SOD activities, and MDA content were significantly (*p* < 0.05) affected by soil water conditions. Results indicated that APX, GR, POD, CAT, and SOD activities were increased and MDA content was decreased by SA, Zn, and GB spraying treatments in both stressed and non-stressed plants (Fig. 3 and Table 3). GR and CAT activities and MDA contents were statistically impacted by concerned spraying treatments in both soil water conditions except for Zn under well-watered condition; while APX, POD, and SOD activities were significantly influenced by concerned spraying treatments under water-deficient condition, and POD by GB application and SOD by SA application under well-watered condition. Under the water-deficient condition, the respective spraying treatments improved the activity of APX by 47.82, 29.89 and 42.39%, GR by 59.62, 42.85 and 48.44%, POD by 26.08, 18.84 and 35.30%, CAT by 62.16, 50.95 and 120.11%, SOD by 61.42, 34.64 and 42.20%, but reduced MDA content by 29.09, 16.64 and 26.51%, respectively, as compared to the values of the control treatment. Overall, the highest APX, GR, and SOD activities and the lowest MDA contents were recorded from the plants treated with SA followed by GB and Zn spraying treatments, while the highest POD and CAT activities were recorded from the plants treated with GB followed by SA and Zn spraying treatments as compared with control treatment under both soil water conditions.

### Reactive oxygen species accumulation

**Fig. 4:**
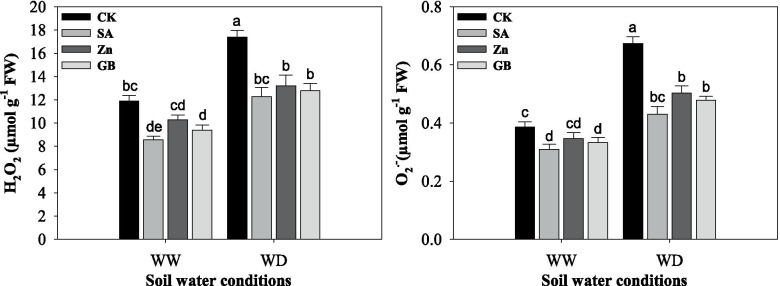
Influences of soil water conditions and foliar treatments on hydrogen peroxide (H_2_O_2_) and superoxide anion (O_2_^.-^) contents. Every column in each graph represents the mean (±SE) of three replicates. Various letters above columns indicate the significant differences among means (*p* < 0.05). WW, well-watered; WD, water-deficient; CK, control (double distilled water); SA, salicylic acid; Zn, zinc; GB, glycine betaine

The level of ROS accumulation was statistically (*p* < 0.05) increased under water-deficient conditions. Results showed that H_2_O_2_ and O_2_^•−^ contents were decreased by SA, Zn, and GB spraying treatments in both stressed and non-stresses plants (Fig. 4 and Table 3). H_2_O_2_ and O_2_^•−^ contents were significantly impacted by concerned spraying treatments under both soil water conditions except for Zn under well-watered conditions. Under the water-deficient condition, the respective spraying treatments decreased the contents of H_2_O_2_ by 29.33, 23.99 and 26.45%, and O_2_^•−^ by 36.10, 25.26 and 28.97%, respectively, as compared to the values of the control treatment. Overall, results indicated that the lowest H_2_O_2_ and O_2_^•−^ contents were recorded from the plants treated with SA followed by GB and Zn spraying treatments as compared with control treatment under both soil water conditions.

### Osmolytes accumulation

**Fig. 5:**
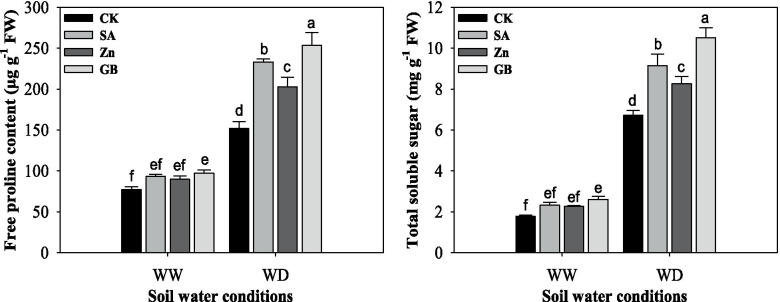
Influences of soil water conditions and foliar treatments on the accumulations of free proline content and total soluble sugar. Every column in each graph represents the mean (±SE) of three replicates. Various letters above columns indicate the significant differences among means (*p* < 0.05). WW, well-watered; WD, water-deficient; CK, control (double distilled water); SA, salicylic acid; Zn, zinc; GB, glycine betaine

Free proline contents and total soluble sugar were significantly (*p* < 0.05) enhanced by the water-deficient condition. Results indicated that free proline contents and total soluble sugar were increased with SA, Zn, and GB spraying treatments in both stressed and non-stressed plants (Fig. 5 and Table 3). Free proline content and total soluble sugar were statistically affected by concerned spraying treatments under water-deficient conditions and GB under well-watered conditions. Under the water-deficient condition, the respective spraying treatments enhanced free proline content by 53.57, 33.41 and 67.07%, and total soluble sugar by 36.01, 22.76 and 56.25%, respectively, as compared to the values of the control treatment. Overall, results showed that the highest free proline and total soluble sugar contents were recorded from the plants treated with GB followed by SA and Zn spraying treatments as compared with control treatment under both soil water conditions.

## Discussion

Drought is a critical agricultural hazard that negatively influences the crop production and may cause a disequilibrium between the defense systems of antioxidant and ROS accumulation, resulting in oxidative damages [6, 8–10, 41–44]. Bayoumi et al. [45] reported that drought led to a decrease in plant height, the number of tillers, 1000-grain weight, spike length, biological and grain yields, and harvest index. Drought stress may hinder the total growth and development of many field crops, and the reproductive growth stages are oversensitive under drought stress [8, 22, 24, 41, 46]. The presented data (Tables 1, 2, and 3) indicated that water-deficient conditions statistically (*p* < 0.05) hampered wheat growth and yield parameters. However, one or more spraying treatments significantly improved growth and yield parameters except for the number of grains spike^− 1^ compared to control treatment in both stressed and non-stressed plants. Previously, plant height and seedling fresh and dry weights were considerably decreased by water deficit, while an exogenous application with GB, Zn, and SA treatments markedly promoted maize growth [22, 25]. In this regard, Anjum et al. [37] and Raza et al. [19] revealed that growth parameters, yield, and yield attributes were statistically decreased by drought stress, while they were significantly increased by exogenous application of GB treatment in maize and wheat plants. Drought stress significantly disrupted the plant height and yield parameters in wheat [8]. Many studies indicated the applications of salicylic acid, zinc, and glycine betaine played a role to regulate protective responses under diverse stresses in many plant species [10, 22, 24–26, 31, 35, 47]. In this study, the impairment in wheat growth performance and yield parameters under water-deficient conditions could be ascribed to excessive production of ROS (H_2_O_2_ and O_2_^•−^) which led to oxidative damage to lipids membrane and raised MDA content (Figs. 3 and 4). In agreement with our results, Mittler [48] and Miller et al. [14] revealed that the increase of ROS accumulation could destroy the cell membrane and lead to immediate damage to lipids, proteins, photosynthetic pigments, nucleic acids, as well as cell structure, and finally caused the death of cells and loss of plant biomass. In contrast to the adverse impacts of the water-deficient condition, drought-tolerance induced by spraying treatments was associated with enhanced contents of *Chl. a*, *Chl. b* and total *Chl.*, and RWC (Fig. 1), improved photosynthesis gas-exchange (Fig. 2), activities of APX, GR, POD, CAT, and SOD, and osmolytes accumulation (Figs. 3 and 5), and decreased the MDA, H_2_O_2_, and O_2_^•−^ contents (Figs. 3 and 4). Growth and yield increase by these exogenous applications under water-deficient conditions is an outside indicator of metabolism alteration in the plant cells. In the past, many studies have indicated the harmful effects of drought stress on the growth performance and yields of various grain crops [10, 11, 22, 46, 49, 50]. However, the damage range under drought stress were differed with the strength of stresses and the crop growth stages [9].

Our results indicated that *Chl. a*, *Chl. b*, and total *Chl.* contents, and RWC were severely decreased in wheat plants by drought stress (Fig. 1). However, SA, Zn, and GB spraying treatments enhanced these parameters in both stressed and non-stressed plants. The decreases in photosynthetic pigments as one of the most substantial restrict factors for plant photosynthetic activity under abiotic stress were documented by numerous other studies [8, 24, 42, 51, 52]. Hussain et al. [11] found that drought stress enhanced the deterioration of chlorophyll in maize plants. In this study, photosynthetic pigments were decreased under water-deficient conditions. Previously, Anjum et al. [37] indicated that the *Chl. a*, *Chl. b* and total *Chl.* contents in maize were reduced under progressive water deficit, while these pigments were substantially increased by GB spray application. However, RWC is a beneficial variable to assess the physiological water status of plant leaves [53]. In this regard, our previous study, Shemi et al. [22] stated that drought stress significantly reduced RWC, whilst exogenous applications with GB, Zn, and SA treatments improved the RWC under drought stress. Bayoumi et al. [45] reported that the RWC was decreased under drought stress in wheat genotypes. Likewise, drought stress at the grain filling phase substantially reduced RWC and chlorophyll content, while exogenous applications of zinc and salicylic acid had a positive impact on these parameters and alleviated the harmful effects of water deficit on wheat plants [20].

In the present study, net photosynthesis rate, transpiration rate, stomatal conductance, and intercellular CO_2_ concentration were considerably affected by soil water condition, while they were increased by SA, Zn, and GB spraying treatments except for intercellular CO_2_ concertation (Fig. 2). Anjum et al. [37] found that the gas-exchange parameters were considerably reduced in maize cultivars under water-stressed conditions, while GB application considerably improved gas-exchange rate under water deficit as compared with control treatment. Shemi et al. [22] found that the net photosynthesis rate, transpiration, and stomatal conductance were decreased by water deficit, while exogenous application of GB, Zn, and SA treatments enhanced the CO_2_ assimilation and improved physiological water status. Under water-deficit conditions, plants tend to close the stomata, which causes reduction in CO_2_ availability and decrease the photosynthetic rate and finally reduces plant yield [54, 55]. In our study, the water-deficient condition significantly increased the APX, GR, POD, CAT, and SOD activities (Fig. 3). However, the increases in activity of antioxidant enzymes were not adequate to protect against ROS accumulations and were not enough to repair the injuries of the oxidative stress triggered by the water-deficient condition. Exogenous applications of salicylic acid, zinc, and glycine betaine to wheat plants exposed to the water-deficient condition increased the activity of these antioxidant enzymes (Fig. 3). Exogenous applications modified the enzymatic and non-enzymatic antioxidants in wheat plants under stress conditions and efficiently quench the harmful ROS (Figs. 3 and 4). In the past, several studies have noticed that antioxidant enzymes and non-enzymes activities were increased, but the contents of H_2_O_2_, O_2_^•−^ and MDA were decreased by exogenous applications of Zn, SA, or GB under drought stress in various plants [10, 19, 22, 28, 31, 35, 47, 56]. In this regard, Miller et al. [14] indicated that diverse enzymatic antioxidants could maintain the equilibrium between production and scavenging of ROS, whereas activities of these enzymes may protect the plants under stress conditions. Furthermore, the foliar application of GB, Zn, and SA treatments substantially enhanced the activity of SOD, CAT, and APX enzymes and scavenged ROS accumulation in maize [22]. The SOD enzyme is considered as the first line for protecting against ROS accumulation, which stimulates the transformation of O_2_^•−^ to O_2_ and H_2_O_2_ [57]. The CAT enzyme stimulates the conversion of H_2_O_2_ to H_2_O and molecular oxygen, where H_2_O_2_ is considered a powerful and harmful oxidizing agent [58]. MDA is regarded as an appropriate indicator for lipid peroxidation in cell membranes [48]. The present study showed that MDA content in wheat leaves was statistically increased under the water-deficient condition and it was well associated with H_2_O_2_ and O_2_^•−^ contents, while respective foliar applications decreased MDA, H_2_O_2_, and O_2_^•−^ contents (Figs. 3 and 4). This finding indicated that exogenous applications raised the ability of plants to cope with oxidative stresses, and therefore improved the drought-tolerance.

The presented results revealed that ROS accumulation (such as H_2_O_2_ and O_2_^•−^ contents) was statistically increased under the water-deficient condition and decreased by SA, Zn, and GB spraying treatments (Fig. 4). This was also proved by Wang and Jin [35], Sofy [48], Talaat et al. [6], Abdel-Motagally and El-Zohri [8], Datir et al. [17], Hasanuzzaman et al. [40] and Shemi et al. [22] who indicated ROS accumulation was considerably increased under drought stress and decreased by exogenous applications of SA, Zn, and GB in various field crops. In this regard, APX and GR are two main enzymes in the cycle of GSH-AsA, that are useful for scavenging H_2_O_2_ and O_2_^•−^ in the compartments of cellular, especially in the chloroplast [6, 24, 42, 43]. In this study, the activity of enzymes was usually promoted by exogenous applications of SA, Zn, and GB as compared to the control treatment. In addition to the enzymatic defense system, some compatible solutes vigorously participate in the amelioration of drought stress. Proline and soluble sugar are very important for the osmoregulation process in plants under drought stress. In this study, proline and soluble sugar in wheat leaves were enhanced under the water-deficient condition and they were also significantly increased by SA, Zn, and GB spraying applications (Fig. 5). This phenomenon can be considered as a portion of the mechanism to inhibit water loss in plants by adjusting the osmotic condition [42, 44]. In our previous study, free proline and soluble sugar in maize were improved by SA, Zn, and GB treatments under drought stress [22]. Furthermore, Hussain et al. [11] and Abdel-Motagally and El-Zohri [8] indicated that proline content and soluble sugar were noticeably enhanced in tested plant leaves under drought stress compared to control treatments. Nasrin et al. [47] revealed that proline and soluble sugar content were significantly increased by the application of salicylic acid under different irrigation schedules. Moreover, El Tayeb and Ahmed [59] remarked that the accumulation of sugars content in shoots and roots of wheat cultivars was substantially promoted under water stress and increased by exogenous application of SA. Proline content was substantially increased under water deficit as compared to well-watered conditions in wheat genotypes [45].

**Fig. 6:**
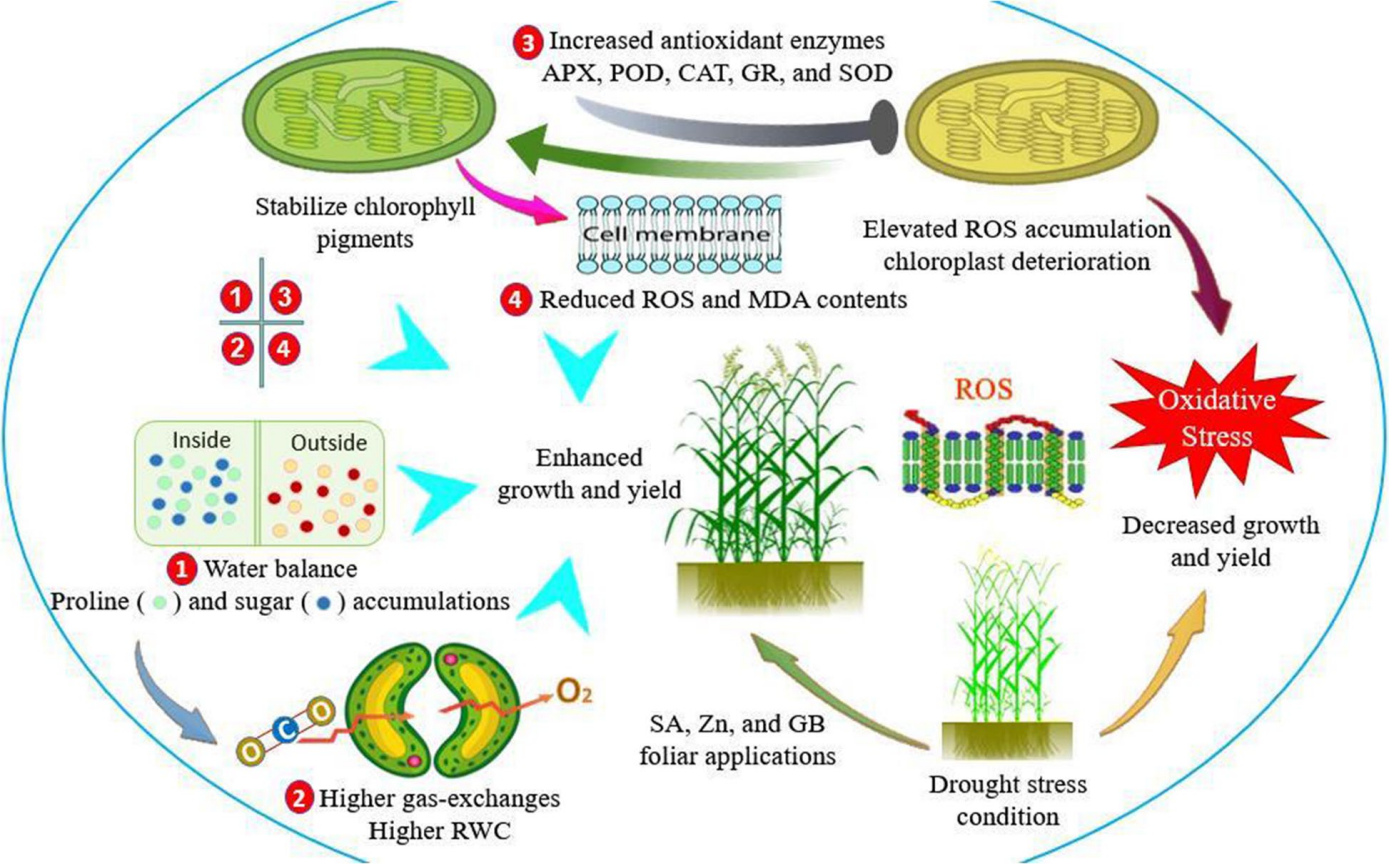
The various mechanisms of foliar salicylic acid (SA), zinc (Zn) and glycine betaine (GB) treatments induced drought-tolerance in wheat plants. RWC, relative water content; APX, ascorbate peroxidase; GR, glutathione reductase; POD, peroxidase; CAT, catalase; SOD, superoxide dismutase; ROS, reactive oxygen species; MDA, malonaldehyde

As noticed in this study, all precedent results proposed that foliar applications could be regarded as protection compounds against oxidative damage caused by drought stress, which could improve the photosynthesis pigments, RWC, leaf gas-exchange, and the capacity of the antioxidant defense system, decrease the MDA, H_2_O_2_, and O_2_^•−^ contents, and increase the osmolyte accumulation in water-stressed plants, thus they might be considered as an important strategy to improve plant growth and yield attributes under drought stress (Fig. 6).

## Conclusions

The present study pointed out that the exogenous application of SA, Zn, and GB treatments could partially mitigate the harmful effects of water stress on the growth of wheat through increasing chlorophyll contents, RWC, modulating gas-exchange traits, enhancing expression of antioxidant enzymes, scavenging ROS, and increasing accumulation of osmolytes. These mechanisms are very important to sustained wheat production in comparatively water-meager regions. Among the foliar application compounds, GB was most effective followed by SA and Zn in promoting the growth and yield attributes of wheat. It could be concluded that exogenous GB, SA, and Zn applications could minimize the loss of wheat yield triggered by drought stress.

## Materials and methods

### Experimental design and plant growth conditions

The controlled pot experiment was conducted during the winter cultivating season of 2018–2019 at the glasshouse of the College of Agronomy and Biotechnology, Southwest University, Chongqing, China. The experimental area lies at longitude 106^°^ 26′ 02″ E, latitude 29^°^ 49′ 32″ N, and altitude 220 m. During the cultivation season, the average minimum and maximum temperatures were 10  to 26 °C, and the relative humidity was between 75 to 87%. The experiment was implemented in a completely randomized design (CRD) with two factors: two soil water conditions (well-watered condition at 85% of field capacity, and water-deficient condition at 50% of field capacity), and four spraying treatments [0.00 double distilled water (CK), 140 mg l^− 1^ salicylic acid (SA), 4 g l^− 1^ zinc (Zn), and 11.5 g l^− 1^ glycine betaine (GB)]. The effective concentrations of these spraying treatments were based on enhancements in the growth and yield of various crops under water-deficient conditions [10, 19–22]. The experiment included eight treatments, and each treatment involved twelve pots. A winter wheat cultivar Yumai-13, which was bred by Sichuan Agricultural University and Chongqing Academy of Agricultural Sciences in China, was used as plant material, and the seeds were obtained from Wheat Research Institute, College of Agronomy and Biotechnology, Southwest University, Chongqing, China.

Each plastic pot (25 cm diameter, 30 cm depth) was filled with 8 kg air-dried and sieved (0.5 mm) soil, which was collected from the experimental station at the College of Agronomy and Biotechnology, Southwest University. Experimental soil was clay loam, and had the following physical and chemical properties: pH, organic matter, electrical conductivity (EC), bulk density, soil water content at field capacity (FC), total N, available phosphorous, and available potassium were 6.25, 12.58 g kg^− 1^, 0.45 ds/m, 1.44 g cm^− 3^, 24.35%, 0.98 g kg^− 1^, 15.53 mg kg^− 1^, and 86.11 mg kg^− 1^, respectively. At the time of soil filling, 2.7 g controlled-release urea (44.6% N), 3.5 g calcium superphosphate (12% P_2_O_5_), 1.5 g potassium chloride (60% K_2_O) were applied for each pot. Fifteen uniform grains were manually sown in the third week of November in pots at depth of 4–5 cm. Thinning was conducted 10 days after germination, and seven uniform seedlings per pot were selected for the subsequent studies. Each pot was irrigated to 85% FC by tap water till the start of drought stress treatments.

### Soil water conditions

The plants were subjected to two soil water conditions for 30 days, from booting (Feekes 10 stage) until milk (Feekes 11 stage) stages of wheat: well-watered condition (85% of field capacity; WW) and water-deficient condition (50% of field capacity; WD). During the drought period, the pots were weighed daily to keep the required soil water levels by adding proper water volumes. Soil water contents for 85 and 50% field capacity were 22.5 and 11.25%, respectively [22]. Soil water content (SWC) was determined using the following equation: SWC % = [(FW-DW)/DW] × 100, where FW was the fresh weight of soil sample from the inner area of each pot, and DW was the dry weight of soil sample after oven drying at 85 °C for 3 days [60].

### Spraying treatments

After 7 and 15 days of drought treatment, the wheat plants under each water condition were sprayed with 0.00 (double distilled water; CK), 140 mg l^− 1^ SA (hydroxybenzoic acid-2 C_7_H_6_O_3_, MW = 138.12 g mol^− 1^), 4 g l^− 1^ Zn (zinc sulfate heptahydrate ZnSO_4_. 7H_2_O, MW = 287.54 g mol^− 1^), and 11.5 g l^− 1^ GB (betaine C_5_H_11_NO_2_, MW = 117.14 g mol^− 1^) [22]. Tween-20 (0.05%) was added with foliar applications as a surfactant at the time of treatment.

### Measurements and analysis

Wheat plants were sampled after 15 days of foliar application treatments to measure growth**,** photosynthetic pigments, RWC, photosynthesis gas-exchange, and biochemical assays. Completely expanded, undamaged, and healthy wheat plant leaves (2^nd^ leaf from the top) from all replicates were sampled. After washing, wheat leaves were frozen with liquid N_2_ immediately and stored at − 80 °C for biochemical analyses, and analysis of yield attributes were recorded at harvesting time.

### Growth, yield, and its attributes

Four pots were randomly selected and plants of each pot were taken to measure plant height, fresh weight pot^− 1^, dry weight pot^− 1^, and leaf area pot^− 1^. Total leaf area pot^− 1^ was measured with LI-3100 leaf area meter (Li-COR, CID, Inc., USA). The dry weight of plants pot^− 1^ was estimated following oven drying at 85 °C for 48 h. At full maturity (plants at 160-days old), five pots were randomly selected, and plants of each pot were harvested to measure the number of tillers pot^− 1^, number of spikes pot^− 1^, number of grains spike^− 1^, grain weight spike^− 1^ (g), 1000-grain weight (g), biological yield pot^− 1^ (g), grain yield pot^− 1^ (g), and harvest index (HI). The HI was computed as the percent ratio of grain yield and biological yield according to Donald [61].

### Photosynthetic pigments and RWC

Chlorophyll (*Chl. a*, *Chl. b*, and total *Chl.*) contents were determined in the 2^nd^ leaf from the top according to Peng and Liu [62]. The extraction of a 200 mg leaf blade sample was done with 10 ml ethanol-acetone (1:2, v/v), and the extract was moved to a 15 ml centrifuge tube. The tubes were put in the dark to avoid light for 24 h until the sample changed into a white color. The chlorophyll content was calculated by the following equation: Chlorophyll a content (mg/g tissue) = (12.7D_663_–2.69D_645_) × V/ (1000 × W), Chlorophyll b content (mg/g tissue) = (22.7 D_645_–4.68D_663_) × V/ (1000 × W), and total chlorophyll (mg/g tissue) = D_652_ × V/ (34.5 × W) / *Chl a* + *Chl b*, where, D_663_, D_645_, and D_652_, respectively are the corresponding wavelengths of the light density value, V is the volume of extracting liquid and W is the weight of fresh leaf. The RWC of wheat leaves was measured according to Barrs and Weatherley [63]. Fresh leaves were cut into small segments (1.5 cm length), weighed fresh weight (FW), then floated in distilled water for 4 h under low light to register saturated weight (SW), and then dried in an oven until constant weight at 80 °C for 24 h to record dry weight (DW). RWC was computed as: RWC = (FW - DW)/ (SW - DW) × 100%.

### Photosynthesis gas-exchange

Net photosynthesis rate, transpiration rate, stomatal conductance, and intercellular CO_2_ concentration were registered using a portable infrared gas analyzer-based photosynthesis system (LI-6400; LiCor, Inc., Lincoln, NE, USA) at 09:30–11:30 am from the fully expanded leaf (2^nd^ leaf from top). Air relative humidity and ambient CO_2_ concentrations were about 78% and 370 μmol CO_2_ mol^− 1^, respectively during the collection of data.

### Assay of enzymatic antioxidants and lipid peroxidation

Antioxidant enzyme activity was reported using commercial kits for glutathione reductase (GR, A111), superoxide dismutase (SOD, A500), catalase (CAT, A501), ascorbate peroxidase (APX, A304), and peroxidase (POD, A502), by following the manufacturer’s instructions (Sino Best Biological Co., Ltd., China). The absorbance readings of GR, SOD, CAT, APX, and POD were detected at 340, 560, 240, 290, and 470 nm, respectively using an ultraviolet (UV)-visible spectrophotometer, and their activities were expressed as units per fresh weight (U g^− 1^ FW). One unit of GR activity was expressed as the amount of enzyme depleting 1 μmol NADPH in 1 min, one unit of SOD activity was defined as the amount of enzyme needed to reduce the reference rate to 50% of maximum inhibition, one unit of CAT activity was measured as the amount of enzyme that decomposes 1 nmol H_2_O_2_ at 240 nm min^− 1^ in 1 g fresh weight, one unit of APX was estimated as the amount of enzyme required for catalyzing 1 μmol ASA at 290 nm 2 min^− 1^ of 1 g fresh weight in 1 ml of a reaction mixture, and one unit of POD activity was demonstrated as the absorbance change of 0.01 at 470 nm min^− 1^ for 1 g fresh weight in 1 ml of a reaction mixture [22]. Lipid peroxidation in wheat leaves was assayed as MDA content, and was measured by thiobarbituric (TBA) method using MDA Detection Kit (A401). Lipid hydroperoxide degradation products could condense with thiobarbituric acid (TBA) to yield red compounds [22]. The absorbance for MDA content was recorded at 532 and 600 nm and expressed as nmol g^− 1^ fresh weight.

### Determination of reactive oxygen species accumulation

The contents of H_2_O_2_ and O_2_^•−^ in the wheat leaves were noted using the commercial ‘H_2_O_2_ Detection Kit (A400)’ and ‘O_2_^•−^ Detection kit (A407)’, respectively, according to the manufacturer’s instructions. H_2_O_2_ content was estimated at 415 nm and represented as μmol g^− 1^ fresh weight. Super oxygen anion serotonin reacted with hydrochloride to produce NO_2_^−^. The NO_2_^−^ interacted with amino benzene and alpha-pyridoxine to produce red compounds at 530 nm which had a characteristic absorption peak [22]. The content of O_2_^•−^ was measured at 530 nm and expressed as μmol g^− 1^ fresh weight.

### Estimation of osmolytes accumulation

Proline and soluble sugar contents in wheat leaves were determined using commercial kits for proline (PRO, A605) and soluble sugar contents (SSC, B602), according to the manufacturer’s instructions (Sino Best Biological Co., Ltd., China). The absorbance reading of the toluene layer was estimated at 520 nm, on a spectrophotometer, and proline (Sigma, St Louis, MO, USA) was used for the standard curve [22]. Proline content was expressed as μg g^− 1^ fresh weight. The absorbance reading of SSC was detected at 620 nm using an ultraviolet (UV)-visible spectrophotometer [22]. Soluble sugar content was articulated as mg g^− 1^ fresh weight.

### Statistical analysis

The collected data were analyzed following the analysis of variance (ANOVA) according to the Two-way Factorial Design using Statistical Software Package MSTAT-C [64]. The significant differences among mean values were estimated according to Least significant difference test (L.S.D.) at a 95% confidence level [65]. Sigma Plot 10.0 (Systat Software Inc., San Jose, CA, USA) was used for the graphical presentation of the data.

## Data Availability

All the data in this work is available in this manuscript and supplementary files. The data that support the findings of this study are available from the corresponding author upon reasonable request.

## Abbreviations

SA: Salicylic acid
Zn: Zinc
GB: Glycine betaine
CK: Double distilled water
RWC: Relative water content
ROS: Reactive oxygen species
H_2_O_2_: Hydrogen peroxide
O_2_^•−^: Superoxide anion radical
MDA: Malonaldehyde
GR: Glutathione reductase
SOD: Superoxide dismutase
CAT: Catalase
APX: Ascorbate peroxidase
POD: Peroxidase
PEG: Polyethylene glycol
*Chl.*: Chlorophyll
Pn: Net photosynthesis rate
Tr: Transpiration rate
Gs: Stomatal conductance
Ci: Intercellular CO_2_ concentration
WW: Well-watered condition
WD: Water-deficient condition
SWC: Soil water content
